# Supplementary figures and images for: Osteoblast-intrinsic defect in glucose metabolism impairs bone formation in type II diabetic mice
Source: bioRxiv. 2023 Jan 18:2023.01.16.524248. Preprint. [Version 1] doi: 10.1101/2023.01.16.524248 (PMC9882117; doi:10.1101/2023.01.16.524248)

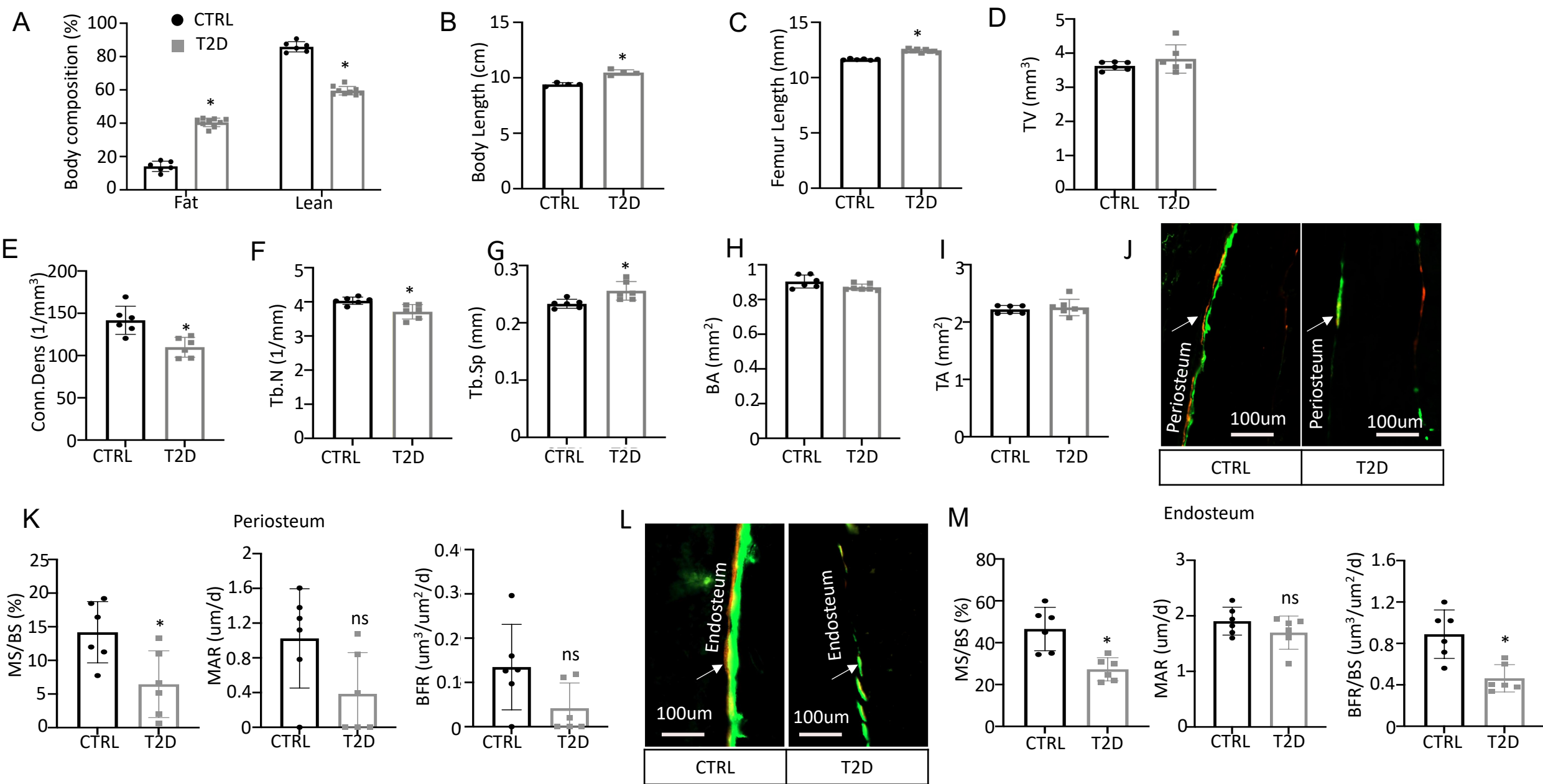

Fig. S1

A

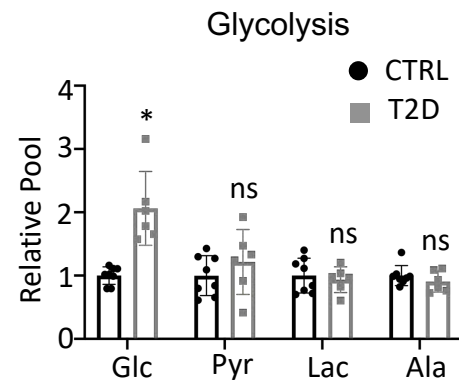

B

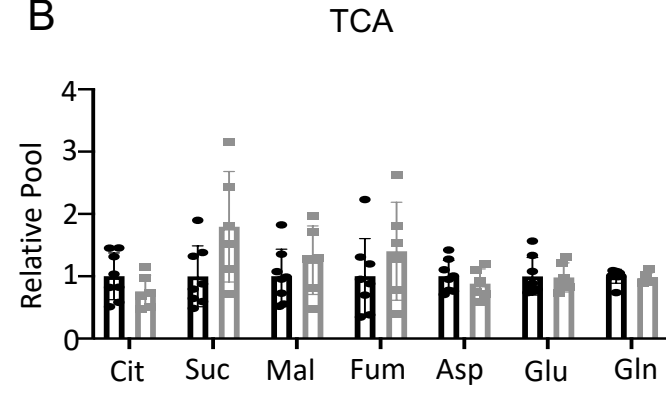

Fig. S2

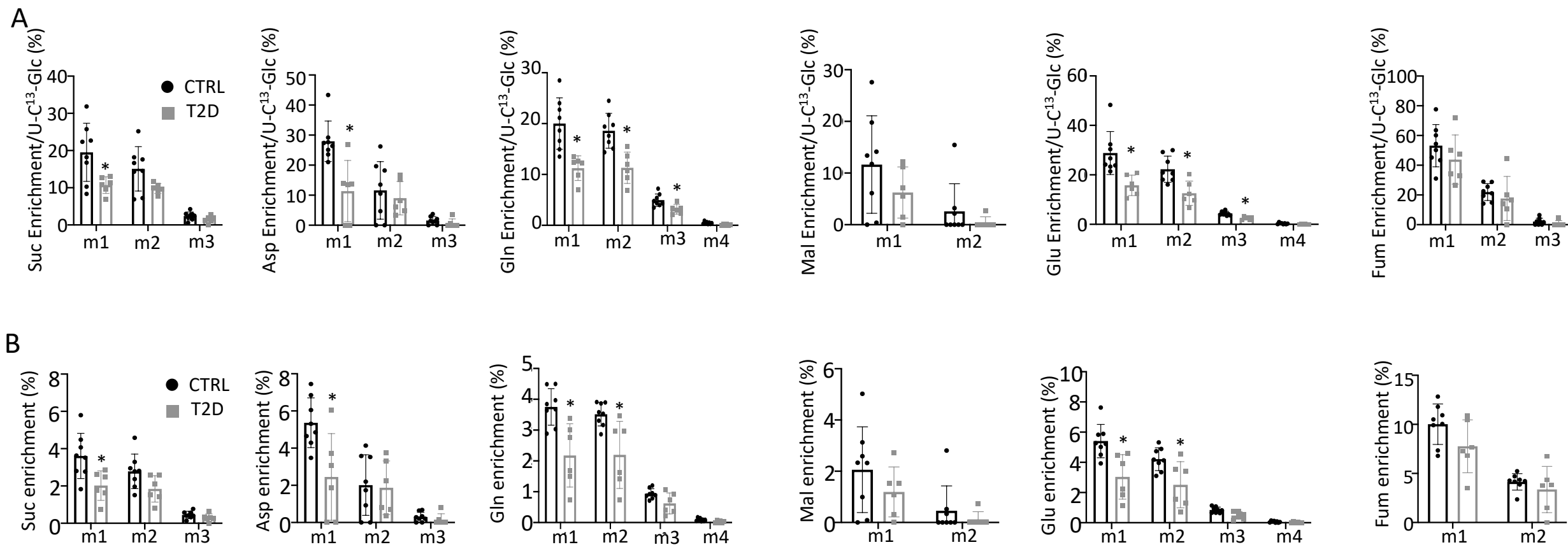

Fig. S3

A

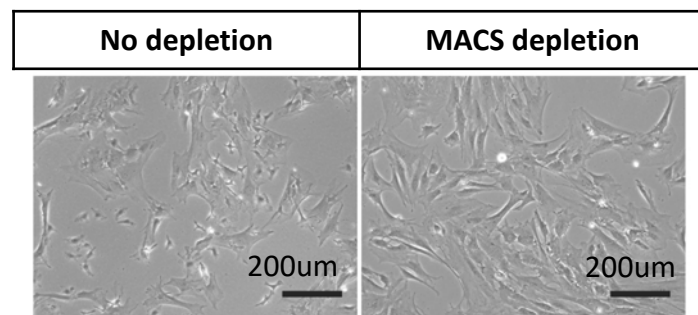

B

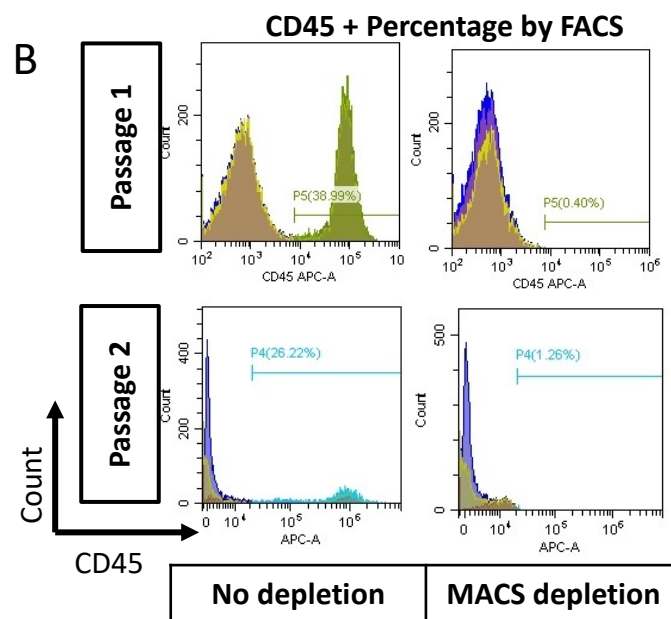

Fig. S4

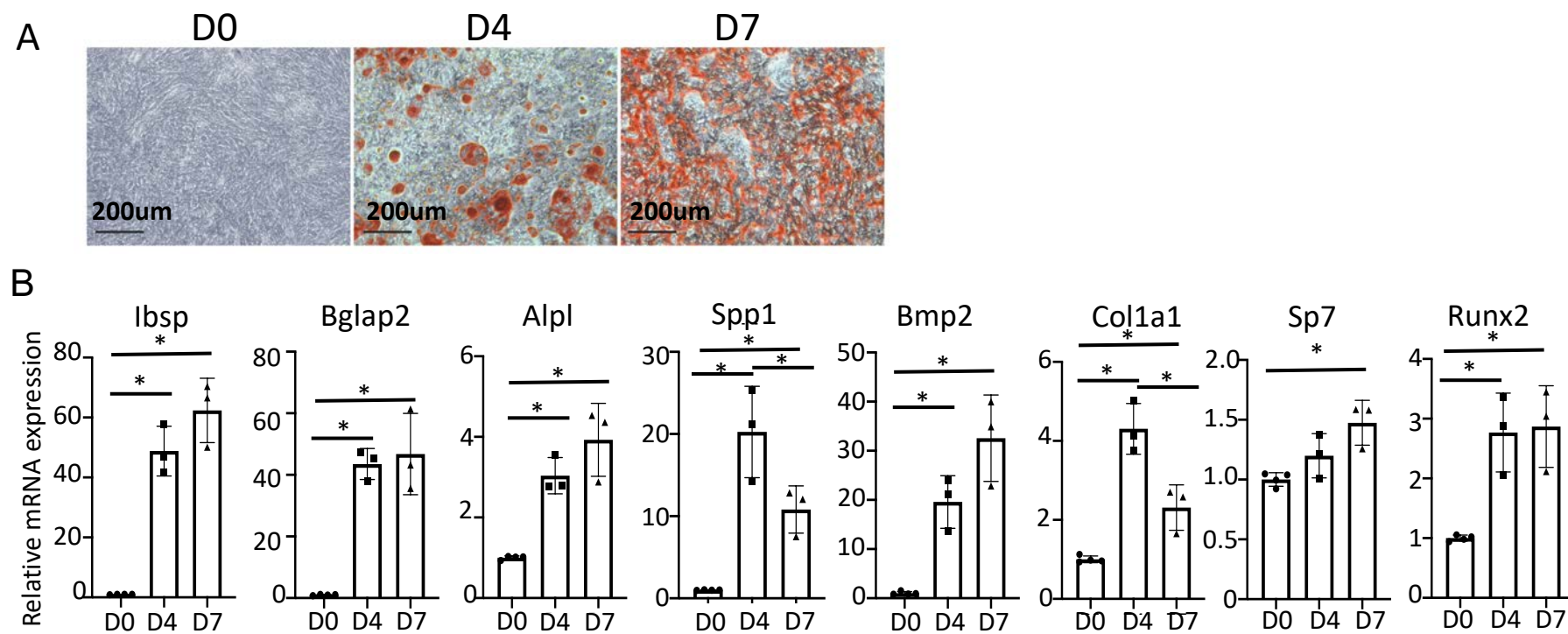

Fig. S5

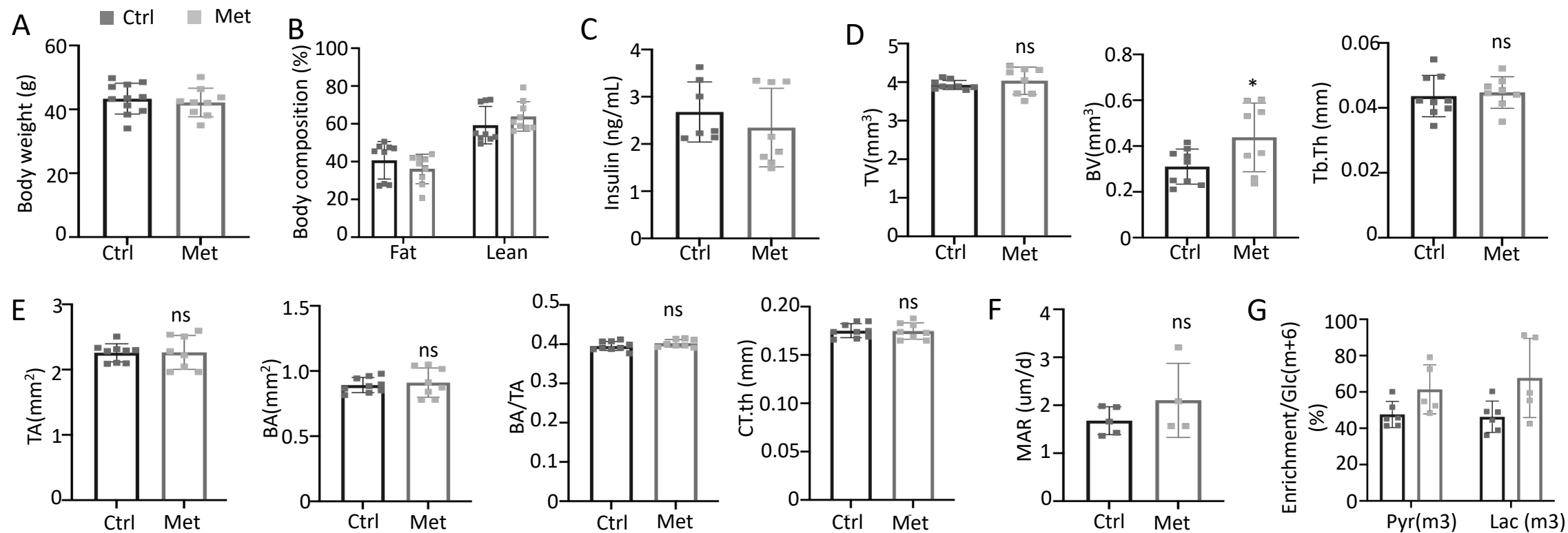

Fig S6

A

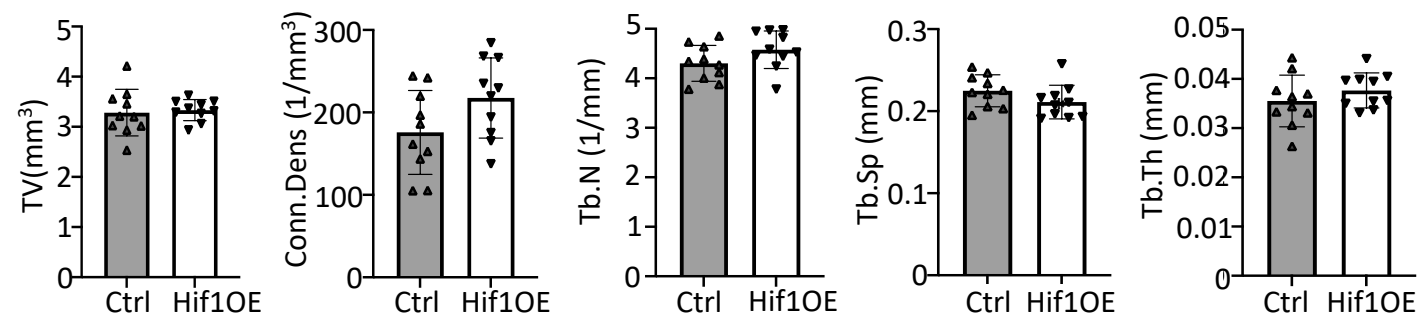

B

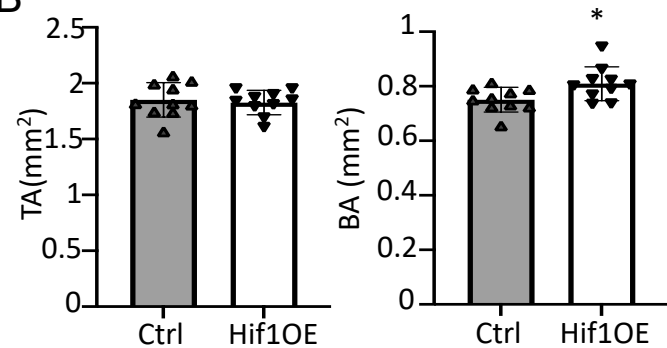

C

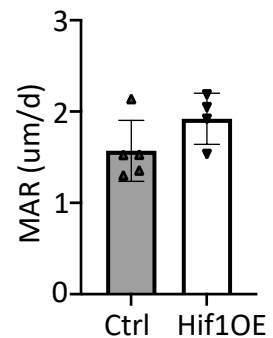

Fig S7

A

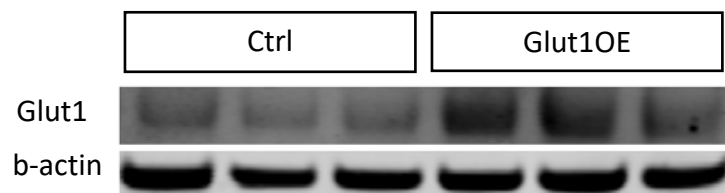

B

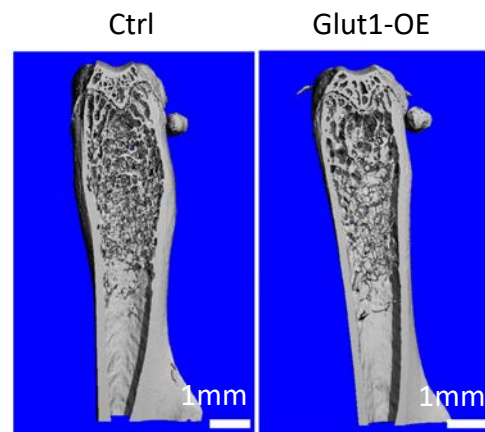

C

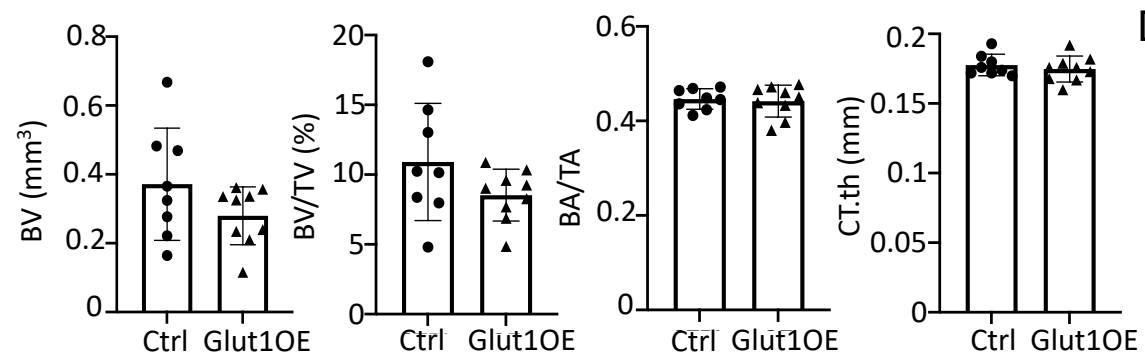

D

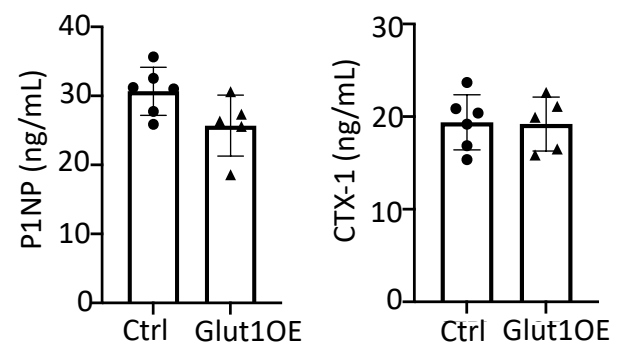

Fig. S8

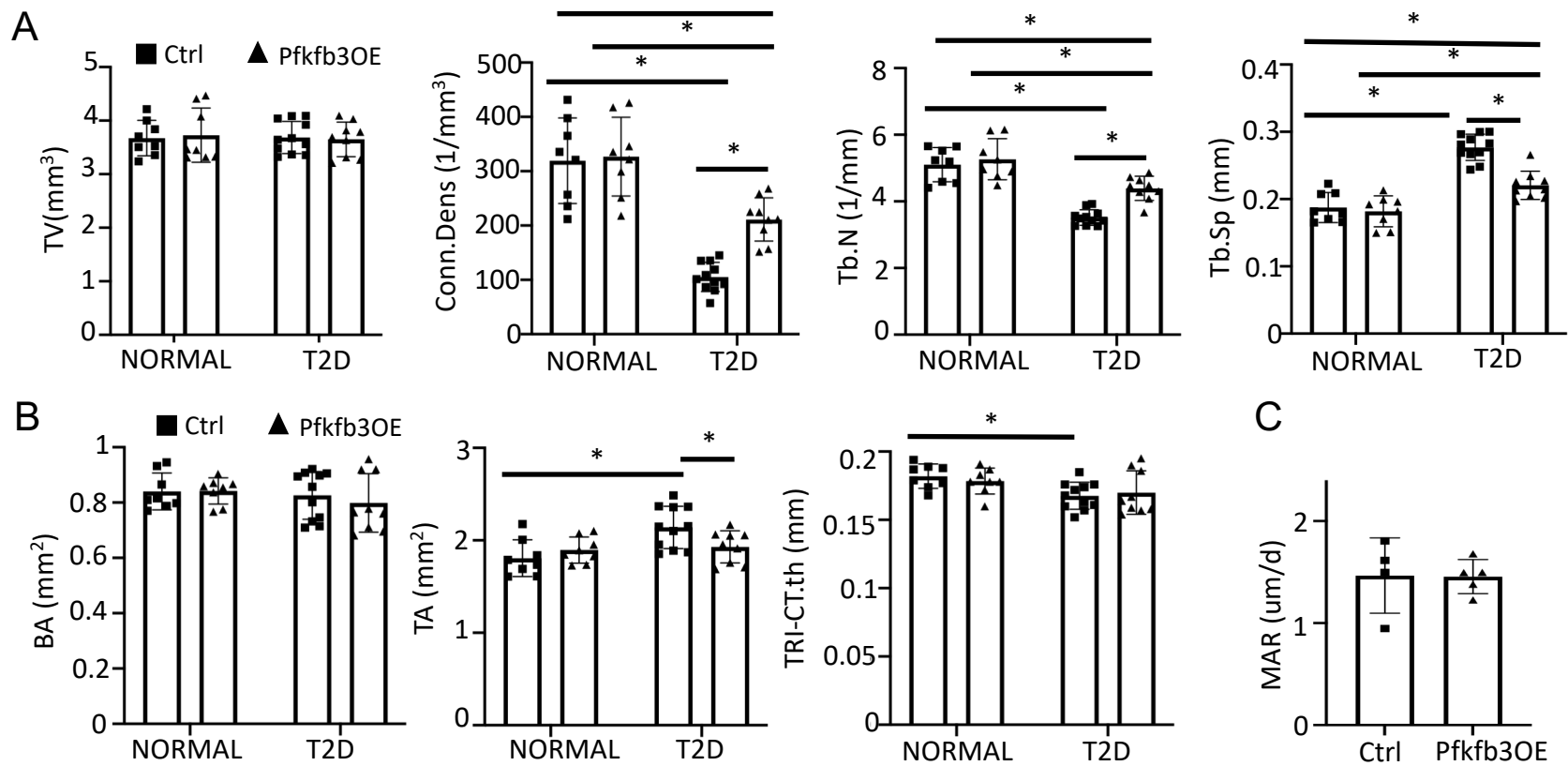

Fig. S9

Supplement: 1 — Figure S1. Diabetic osteopenia in T2D mouse model. (A) Body composition. CTRL, n=6; T2D, n=9. (B) Body length (cm) from nose tip to anus. n=4. (C) Femur length measured by DXA. CTRL, n=6; T2D, n=9. (D-G) Trabecular bone parameters by μCT. n=6. (H) Cortical bone parameters by μCT. CTRL, n=6; T2D, n=7. (J-K) Double labeling analysis of periosteum. n=6. (L-M) Double labeling analysis of endosteum. n=6. Data are represented as mean ± SD. *P < 0.05 as determined by Student’s t test between two groups. Figure S2. Glucose tracing in plasma. (A, B) Relative levels of glycolysis (A) and TCA (B) metabolites in the plasma. CTRL, n=8; T2D, n=6. Data are represented as mean ± SD. *P < 0.05, Student’s t test. Figure S3. Glucose tracing in bone. (A) Relative enrichment of specific isotopomers normalized to 13C6-Glc in bone. (B) Enrichment of specific isopotomers in bone. CTRL, n=8; T2D, n=6. Data are represented as mean ± SD. *P < 0.05, two-way ANOVA followed by Sidak’s multiple comparisons. Figure S4. BMSC purification with MACS beads. (A) Bright field of BMSC with or without depletion of CD45+ cells. (B) FACS analysis of CD45+ cell percentage for BMSC with or without depletion after one or two passages. Figure S5. In vitro osteoblast differentiation of MACS-purified BMSC from wild type mice. (A) Alizarin red staining with differentiation at day 0, day 4 and day 7. (B) qPCR of osteoblast marker genes. n=3. Data are represented as mean ± SD. *P < 0.05 by one-way ANOVA followed by Student’s t test. Figure S6. Metformin improves bone mass in T2D mice. (A) Body weight. Ctrl, n=11; Met, n=9. (B) Body composition. n=9. (C) Serum insulin level. Ctrl, n=7; Met, n=8. (D) Trabecular bone parameters of femurs by μCT. Ctrl, n=9; Met, n=8. (E) Cortical bone parameters of femur midshaft. Ctrl, n=9; Met, n=8. (F) Mineral apposition rate by double labeling method. Ctrl, n=5; Met, n=4. (G) Relative enrichment of specific isotopomers normalized to Glc(m+6). Ctrl, n=6; Met, n=5. Data are represente [file NIHPP2023.01.16.524248v1-supplement-1.pdf]
